# Prognostic value of thrombocytopenia during hospitalizations in intracerebral hemorrhage patients

**DOI:** 10.3389/fneur.2024.1429988

**Published:** 2024-11-22

**Authors:** Hao Feng, Xiaoquan Luo, Anhui Fu, Ruoran Wang, Fei Qiao

**Affiliations:** ^1^Department of Neurosurgery, Nanchong Central Hospital/The Second Clinical Medical College, North Sichuan Medical College, Nanchong, China; ^2^Department of Neurosurgery, West China Hospital, Sichuan University, Chengdu, China

**Keywords:** intracerebral hemorrhage, thrombocytopenia, platelet, mortality, hemorrhagic stroke

## Abstract

**Background:**

The thrombocytopenia influences prognoses of critically ill patients. There are few studies exploring the prognostic value of thrombocytopenia among ICH patients. We perform this study to explore the correlation between thrombocytopenia at different timepoints of hospitalizations and mortality of ICH.

**Methods:**

ICH patients recorded in the Medical Information Mart for Intensive Care-III were selected for this observational study. The thrombocytopenia, defined as platelet <150 × 10^9^/L, was divided into three categories: baseline thrombocytopenia (thrombocytopenia occurred at admission), acquired thrombocytopenia (thrombocytopenia developed since the second day after admission), multiple thrombocytopenia (baseline thrombocytopenia + acquired thrombocytopenia). The main outcome in this study was the 30-day mortality of ICH patients. The univariate and multivariate logistic regression was sequentially performed to discover risk factors of mortality and confirm the correlation between thrombocytopenia groups and mortality of ICH.

**Results:**

66.5% of 902 ICH patients did not experience the thrombocytopenia since admission. 2.2, 14.5 and 16.7% ICH patients showed the baseline thrombocytopenia, acquired thrombocytopenia initial and multiple thrombocytopenia, respectively. The GCS did not show significant difference between thrombocytopenia groups (*p* = 0.118). The multiple thrombocytopenia group had the highest incidence of mechanical ventilation (*p* = 0.041), mortality (*p* < 0.001), and the longest length of ICU stay (*p* < 0.001), length of hospital stay (*p* < 0.001). The multivariate logistic regression found age (*p* < 0.001), GCS (*p* < 0.001), glucose (*p* = 0.013), mechanical ventilation (*p* = 0.002) was correlated with the mortality of ICH patients. Only the multiple thrombocytopenia group showed significant influence on the mortality of ICH (*p* = 0.002) in the multivariate logistic regression.

**Conclusion:**

Single initial thrombocytopenia at admission dose not influence the mortality of ICH patients. ICH patients experiencing both initial thrombocytopenia and acquired thrombocytopenia have significantly higher mortality risk. The blood platelet level of ICH patients should be monitored continuously during hospitalizations to detect the thrombocytopenia and identify the high risk of poor prognosis.

## 1 Introduction

Accounting for nearly one third of stroke incidence, the intracerebral hemorrhage (ICH) leads poor prognosis of patients with the mortality more than 50% (1, 2). As the essential component of coagulation and hemostasis, the platelet plays an important role on the pathophysiological process of stroke. Both abnormalities of platelet function and number significantly may influence the bleeding events and prognoses of stroke patients (3, 4). The thrombocytopenia, commonly defined as the platelet level < 150 × 10^9^/L, has been confirmed correlated with a serious of bleeding events, transufsion requirements and outcomes of critially ill patients (5, 6). While two studies, respectively, found the thrombocytopenia at admission was not related with the poor outcomes of ICH patients and ichemic stroke patients (7, 8). Another study discovered that only thrombocytopenia developed during ICU stay but not thrombocytopenia at admission was an indepdent risk factor for mortality of patients treated in the neurocritical care unit (9). And one recent study found the risk of hospital mortality in critically ill patients increased significantly with persistent thrombocytopenia but not transient thrombocytopenia (10). These findings lead us to make a hypothesis that thrombocytopenia at different timepoints since admission may exert different effects on prognoses of ICH patients. Therefore, we design this study to explore the prognostici value of thrombocytopenia at admission and thrombocytopenia developed during hospitalizations among ICH patients.

## 2 Materials and methods

### 2.1 Enrolled participants

ICH patients (confirmed using ICD-9 code-431) recorded in the Medical Information Mart for Intensive Care-III (MIMIC-III) (an intensive care database enrolling patients from the Beth Israel Deaconess Medical Center between 2001 and 2012) were selected for this observational study. A part of ICH patients were sequentially excluded from this study for four causes: (1) no records of platelet (*n* = 14); (2) no records of platelet at admission (*n* = 6); (3) records of platelet after day 1 ≤ 2 (*n* = 435); (4) no records of Glasgow Coma Scale (GCS) (*n* = 9). Nine hundred and two ICH patients were enrolled after the screening. The MIMIC-III deidentified personal information of patients and received ethical approvements from the Massachusetts Institute of Technology and Beth Israel Deaconess Medical Center.

### 2.2 Variables collection

Demographic information (age, gale gender), comorbidities (hypertension, hyperlipidemia, diabetes, atrial fibrillation, coronary heart disease, chronic renal disease), vital signs at admission (mean blood pressure, heart rate), pulse oxygen saturation (SpO_2_) and GCS at admission were recorded. Laboratory examinations including white blood cell, hemoglobin, hematocrit, platelet, glucose, calcium, phosphate, prothrombin time, activated partial thromboplastin time, international normalized ratio (INR) were analyzed using the first blood sample after admission (within 24 h since admission). The subsequent platelet level after day 1 was also collected with the median frequency of 9 for measurements. The thrombocytopenia, defined as platelet <150 × 10^9^/L, was divided into three categories: baseline thrombocytopenia (thrombocytopenia occurred at admission), acquired thrombocytopenia (thrombocytopenia developed since the second day after admission), multiple thrombocytopenia (baseline thrombocytopenia + acquired thrombocytopenia). The main outcome in this study was the 30-day mortality of ICH patients. The incidence of mechanical ventilation, length of ICU stay, length of hospital stay was also compared between different thrombocytopenia groups.

### 2.3 Statistical analyses

Variables with normal distribution or non-normal distribution were, respectively presented as mean ± standard or median (interquartile range). Kolmogorov–Smirnov test was used for testing the normal distribution of variables. Patients were divided into different groups according to the occurrence and timepoint of thrombocytopenia. Differences between these groups were compared using one way ANOVA (variables with normal distribution), Kruskal–Wallis (variables with non-normal distribution), or chi-square test (categorical variables). The univariate and multivariate logistic regression was sequentially performed to discover risk factors of mortality and confirm the correlation between thrombocytopenia groups and mortality of ICH. Two sides *p*-value <0.05 was set as statistically significant. All analyses and figures were performed using GraphPad Prism (GraphPad Software Inc., La Jolla, CA, United States) and SPSS 23.0 (SPSS, Inc., Chicago, IL).

## 3 Results

### 3.1 Comparison between thrombocytopenia groups of ICH patients

**Figure 1:**
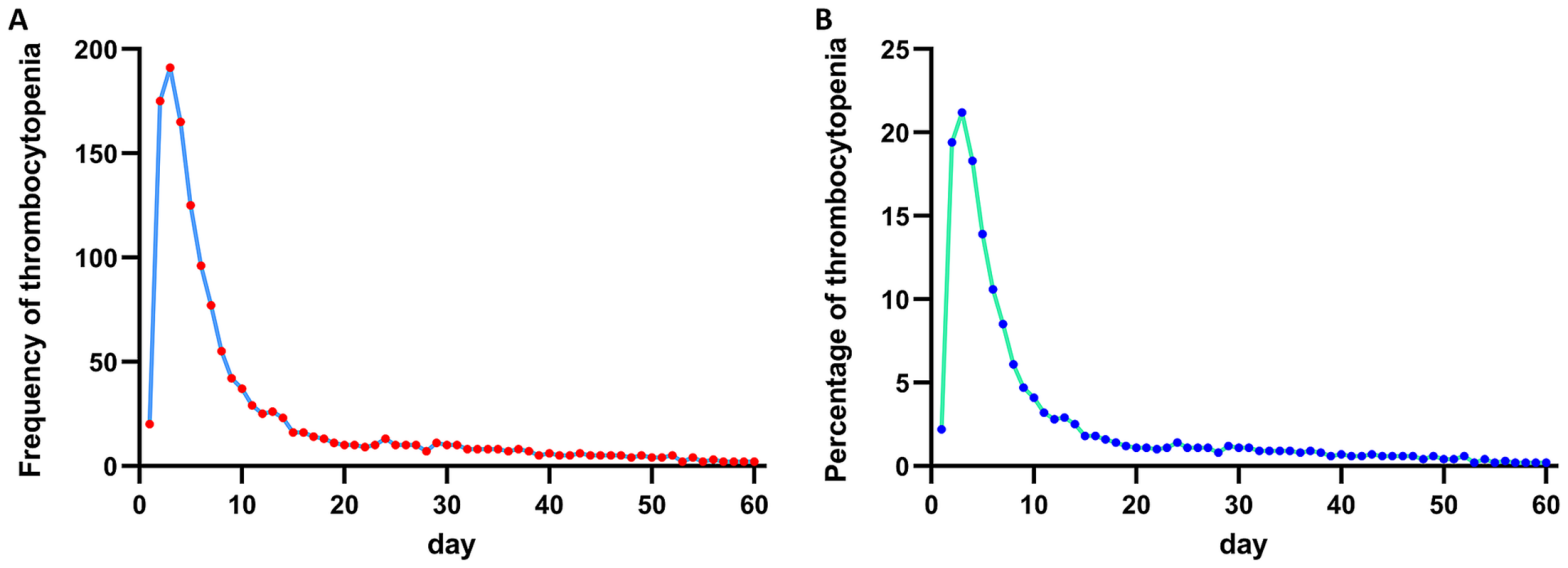
(A) Frequency of thrombocytopenia during hospitalizations in ICH patients. (B) Percentage of thrombocytopenia during hospitalizations in ICH patients.

The occurrence of thrombocytopenia is concentrated in the first 10 days with the peak on the third day since admission (Figures 1A,B). Among 902 ICH patients, 66.5% (*n* = 600) of them did not experience the thrombocytopenia since admission (Table 1). 2.2% (*n* = 20) ICH patients showed the only initial thrombocytopenia at admission and 14.5% (*n* = 131) experienced acquired thrombocytopenia since the second day after admission. 16.7% (*n* = 151) ICH patients experienced both initial thrombocytopenia and acquired thrombocytopenia during hospitalizations (Table 1). Age (*p* = 0.002) and gender ratio (*p* = 0.006) showed significant difference among thrombocytopenia groups. The comorbidity atrial fibrillation was most prevalent in the baseline thrombocytopenia group (*p* = 0.029). The GCS did not show significant difference between thrombocytopenia groups though the multiple thrombocytopenia group had relatively lower GCS [9 (6–14)] (*p* = 0.118). Laboratory examination presented the multiple thrombocytopenia group had the lowest level of hemoglobin (*p* = 0.006), hematocrit (*p* = 0.003), platelet (*p* < 0.001), calcium (*p* < 0.001) and the highest level of prothrombin time (*p* < 0.001), activated partial thromboplastin time (*p* < 0.001), INR (*p* < 0.001). Furthermore, the multiple thrombocytopenia group had the highest incidence of mechanical ventilation (*p* = 0.041), mortality (*p* < 0.001) and the longest length of ICU stay (*p* < 0.001), length of hospital stay (*p* < 0.001).

**Table 1:** Comparison between groups of ICH patients divided by the thrombocytopenia.

	All ICH patients (*n* = 902)	Non-thrombocytopenia (*n* = 600, 66.5%)	Baseline thrombocytopenia (*n* = 20, 2.2%)	Acquired thrombocytopenia (*n* = 131, 14.5%)	Multiple thrombocytopenia (*n* = 151, 16.7%)	*p*
Age (year)	69.2 (56.7–80.0)	67.1 (55.3–79.0)	73.1 (67.9–81.7)	71.4 (61.8–80.5)	72.9 (59.5–82.1)	0.002
Male gender (%)	509 (56.4%)	328 (54.7%)	13 (65.0%)	65 (49.6%)	103 (68.2%)	0.006
Comorbidities						
Hypertension (%)	580 (64.3%)	390 (65.0%)	14 (70.0%)	84 (64%)	92 (60.927%)	0.762
Hyperlipidemia (%)	152 (16.9%)	98 (16.3%)	3 (15.0%)	29 (22.1%)	22 (14.6%)	0.342
Diabetes (%)	193 (21.4%)	123 (20.5%)	6 (30.0%)	33 (25.2%)	31 (20.5%)	0.502
Atrial fibrillation (%)	210 (23.3%)	123 (20.5%)	7 (35.0%)	34 (26.0%)	46 (30.5%)	0.029
Coronary heart disease (%)	148 (16.4%)	91 (15.2%)	5 (25.0%)	27 (20.6%)	25 (16.6%)	0.329
Chronic renal disease (%)	72 (8.0%)	41 (6.8%)	2 (10.0%)	10 (7.6%)	19 (12.6%)	0.135
Vital signs on admission						
Mean blood pressure (mmHg)	94.0 (82.7–105.0)	94.3 (82.7–105.7)	97.7 (85.0–107.0)	92.7 (80.3–103.7)	92.3 (84.7–104.3)	0.830
Heart rate (min^−1^)	79 (69–91)	78 (69–90)	78 (65–84)	82 (72–98)	81 (71–93)	0.007
SpO_2_ (%)	99 (97–100)	99 (97–100)	99 (96–100)	99 (97–100)	99 (97–100)	0.975
GCS	11 (6–15)	12 (6–15)	11 (6–14)	11 (6–15)	9 (6–14)	0.118
Laboratory tests						
White blood cell (10^9^/L)	10.10 (7.90–13.20)	10.60 (8.50–13.50)	7.70 (5.80–12.10)	10.00 (7.80–13.80)	8.40 (5.80–11.10)	<0.001
Hemoglobin (g/dL)	13.0 ± 2.0	13.1 ± 1.9	12.7 ± 1.8	12.9 ± 2.0	12.5 ± 2.1	0.006
Hematocrit (%)	38.0 ± 5.4	38.4 ± 5.2	37.3 ± 4.5	38.0 ± 5.7	36.6 ± 5.9	0.003
Platelet (10^9^/L)	229 (178–285)	256 (216–310)	149 (137–159)	197 (175–246)	133 (92–159)	<0.001
Glucose (mg/dL)	136 (114–169)	134 (114–165)	148 (112–175)	141 (114–189)	141 (115–169)	0.615
Calcium	8.9 (8.4–9.3)	8.9 (8.5–9.4)	8.8 (8.5–9.2)	8.8 (8.4–9.2)	8.6 (8.2–9.0)	<0.001
Phosphate	3.2 (2.7–3.7)	3.2 (2.7–3.7)	3.0 (2.8–3.7)	3.3 (2.8–3.8)	3.0 (2.5–3.6)	0.148
Prothrombin time (s)	13.0 (12.4–14.7)	12.9 (12.2–14.1)	13.5 (13.1–16.6)	13.1 (12.3–14.1)	13.9 (12.7–17.0)	<0.001
Activated partial thromboplastin time (s)	26.4 (23.9–30.0)	26.1 (23.8–29.4)	28.3 (25.6–31.1)	25.9 (23.4–28.6)	28.6 (25.0–33.0)	<0.001
INR	1.1 (1.0–1.3)	1.1 (1.0–1.3)	1.2 (1.1–1.6)	1.1 (1.0–1.3)	1.2 (1.1–1.7)	<0.001
Antiplatelets (%)	604 (67.0%)	402 (67.0%)	13 (65.0%)	94 (71.8%)	95 (62.9%)	0.070
Anticoagulants (%)	195 (21.6%)	121 (20.0%)	5 (25.0%)	40 (30.5%)	29 (19.2%)	0.470
Mechanical ventilation (%)	448 (49.7%)	280 (46.7%)	9 (45.0%)	70 (53.4%)	89 (58.9%)	0.041
Neurosurgical intervention (%)	285 (31.6%)	200 (33.3%)	2 (10.0%)	37 (28.2%)	(30.5%)	0.075
30-day mortality (%)	187 (20.7%)	102 (17.0%)	4 (20.0%)	29 (22.1%)	52 (34.4%)	<0.001
Length of ICU stay (day)	5 (2–10)	4 (2–8)	4 (2–6)	6 (3–12)	6 (3–12)	<0.001
Length of hospital stay (day)	11 (7–17)	10 (6–16)	12 (8–19)	12 (8–20)	13 (8–19)	<0.001

SpO_2_, pulse oxygen saturation; GCS, Glasgow Coma Scale; INR, international normalized ratio.

### 3.2 Correlation between thrombocytopenia and 30-day mortality of ICH patients

**Figure 2:**
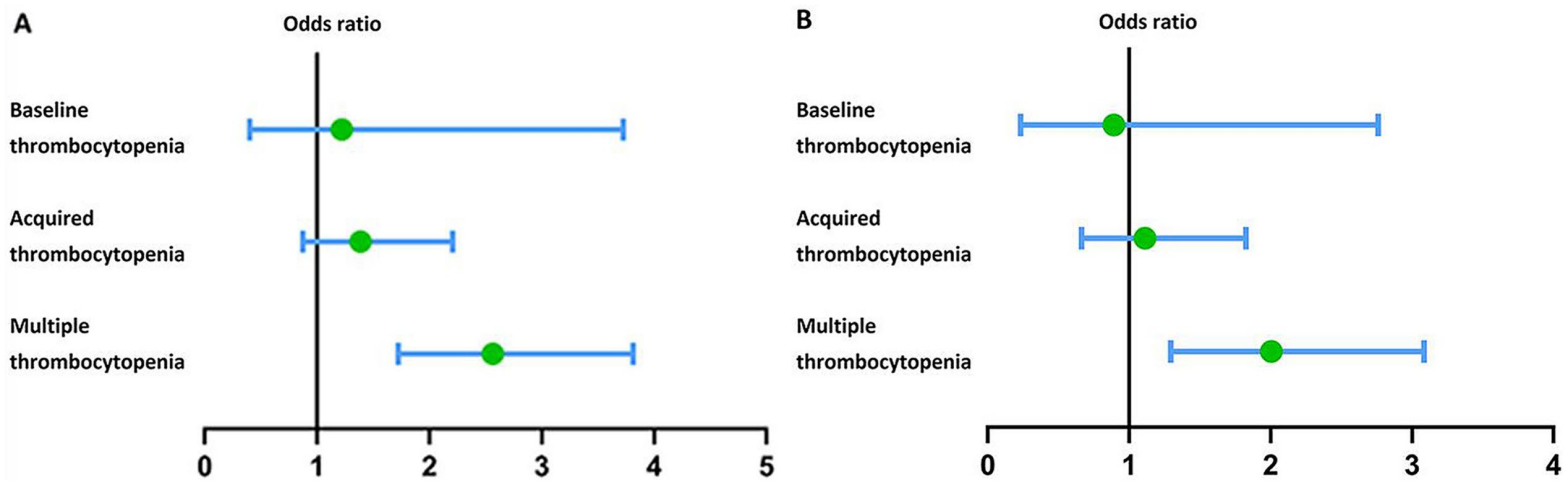
(A) Forest plot showing the correlation between different types of thrombocytopenia and mortality of ICH patients analyzed by the univariate logistic regression. (B) Forest plot showing the correlation between different types of thrombocytopenia and mortality of ICH patients analyzed by the multivariate logistic regression.

The univariate logistic regression presented age (*p* < 0.001), atrial fibrillation (*p* = 0.005), chronic renal disease (*p* = 0.016), mean blood pressure (*p* = 0.021), GCS (*p* < 0.001), hemoglobin (*p* = 0.001), hematocrit (*p* = 0.002), glucose (*p* < 0.001), mechanical ventilation (*p* < 0.001) was correlated with the mortality of ICH patients (Table 2). Only the multiple thrombocytopenia group (*p* < 0.001) showed statistical significance on influencing the mortality of ICH in the univariate logistic regression (Figure 2A). The multivariate logistic regression found age (*p* < 0.001), GCS (*p* < 0.001), glucose (*p* = 0.013), mechanical ventilation (*p* = 0.002) was correlated with the mortality of ICH patients. The multiple thrombocytopenia still showed statistical significance (*p* = 0.002) in the multivariate logistic regression while other thrombocytopenia groups did not correlate with the mortality of ICH patients (Figure 2B).

**Table 2:** Univariate and multivariate logistic regression analysis of correlation between thrombocytopenia and 30-day mortality of intracerebral hemorrhage patients.

Variables	Univariate logistic regression			Multivariate logistic regression		
	OR	95% CI	*p*-value	OR	95% CI	*p*-value
Age	1.036	1.024–1.049	<0.001	1.043	1.028–1.059	<0.001
Male gender	0.793	0.574–1.095	0.158			
Hypertension	0.884	0.634–1.233	0.467			
Hyperlipidemia	1.073	0.702–1.641	0.744			
Diabetes	1.082	0.734–1.594	0.691			
Atrial fibrillation	1.665	1.164–2.382	0.005	1.458	0.935–2.273	0.097
Coronary heart disease	1.016	0.658–1.567	0.944			
Chronic renal disease	1.906	1.129–3.219	0.016	1.890	1.030–3.468	0.040
Mean blood pressure	0.989	0.980–0.998	0.021	0.993	0.983–1.004	0.209
Heart rate	1.009	1.000–1.019	0.051			
SpO_2_	1.015	0.958–1.075	0.610			
GCS	0.872	0.839–0.907	<0.001	0.899	0.850–0.95	<0.001
White blood cell	1.021	0.996–1.046	0.094			
Hemoglobin	0.868	0.799–0.943	0.001	0.890	0.618–1.281	0.530
Hematocrit	0.954	0.926–0.983	0.002	1.004	0.882–1.143	0.951
Glucose	1.005	1.002–1.008	<0.001	1.004	1.001–1.007	0.014
Calcium	0.833	0.681–1.017	0.073			
Phosphate	1.067	0.919–1.240	0.394			
Thrombocytopenia group						0.041
Non-thrombocytopenia	1.000	Reference		1.000	Reference	
Baseline thrombocytopenia	1.221	0.400–3.727	0.726	0.876	0.257–2.991	0.833
Acquired thrombocytopenia	1.388	0.873–2.208	0.166	1.142	0.677–1.926	0.620
Multiple thrombocytopenia	2.564	1.723–3.816	<0.001	1.890	1.215–2.939	0.005
Prothrombin time	1.022	0.999–1.046	0.057			
Activated partial thromboplastin time	1.017	1.000–1.034	0.056			
INR	1.115	0.990–1.256	0.074			
Antiplatelets	0.637	0.415–0.977	0.039	0.520	0.315–0.859	0.011
Anticoagulants	0.639	0.459–0.890	0.008	0.427	0.284–0.643	<0.001
Mechanical ventilation	3.128	2.206–4.434	<0.001	2.545	1.580–4.101	<0.001
Neurosurgical intervention	0.997	0.705–1.410	0.988	1.029	0.677–1.566	0.892

OR, odds ratio; CI, confidence interval; SpO_2_, pulse oxygen saturation; GCS, Glasgow Coma Scale; INR, international normalized ratio.

## 4 Discussion

As a disorder of coagulative system, the thrombocytopenia is prevalent among hospitalized patients with the incidence ranging from 8.3 to 67.6% (6). Some studies have explored risk factors for the thrombocytopenia including age, gender, sepsis, liver dysfunction, bleeding, disease severity reflected by the sequential organ failure score (6, 11, 12). While the effect of thrombocytopenia on the prognosis of various patients has not been confirmed. Some studies found the thrombocytopenia were related with the mortality risk of various patients including sepsis, COVID-19, seasonal influenza, systemic lupus erythematosus, cancer (12–18). While one study showed the thrombocytopenia at admission was not related with poor outcomes of ichemic stroke patients (8). Another study implied that the thrombocytopenia did not influence rates of hematoma enlargement and functional status of ICH patients, regardless of the prior antiplatelet therapy (7). It is noteworthy that only thrombocytopenia developed during ICU stay but not thrombocytopenia at admission was confirmed an indepdent risk factor for mortality of patients treated in the neurocritical care unit (9). This fact indicated that the thrombocytopenia at different timepoints since admission may exert different effects on prognoses of crtically ill patients. Consistent with the finding, results of our study implied ICH patients complicated with thrombocytopenia both at admission and developed during hospitalizations had significanly higher mortality while the ICH patients with thrombocytopenia only at admission did not show higher mortaliy risk than non-thrombocytopenia ICH patients.

The insignificance of baseline thrombocytopenia indicated that it may just act as a reflection of initial stroke severity or prior use of antiplatelets. The transient effect of low platelet level at admission may not play a critical role on the prognoses of ICH patients. Only our stated multiple thrombocytopenia which meant persistent thrombocytopenia lasting from the admission to subsequent hospitalization significantly influence the mortality of ICH patients. This finding was consistent with one previous study showing that the mortality of critically ill patients begins to significantly increase only when the thrombocytopenia lasts more than 2 days and the mortality is positively associated with the duration of thrombocytopenia (10). The persistent or multiple thrombocytopenia could be caused by many factors including blood dilution, decreased platelet production, increased platelet consumption, and drugs (19, 20). As for ICH patients, the excessive consumption caused by coagulation, intraoperative blood loss and intracranial progressive hemorrhage may both contribute to the persistent state of low platelet level.

Due to the prognostic effect of multiple thrombocytopenia, the platelet level after ICH should be monitored constantly especially during the first 10 days of hospitalizations. And preventive measures for the trend of reduced platelet level including appropriate platelet transfusion should be provided after ICH. Certainly, the detailed schedule and indications of platelet transfusion for ICH patients has not been decided though studies have been performed to validate the efficiency and safety of platelet transfusion in ICH patients especially those with prior antiplatelet use (21–26). Though most of available evidence showed no benefit of platelet transfusion for ICH patients with prior antiplatelet use, the efficacy of platelet transfusion for the general ICH patients receiving surgical intervention has not been confirmed. The discovered effect of thrombocytopenia at different timepoints in our study may be helpful for researchers to design clinical trials exploring the indication of platelet transfusion among ICH patients in the future.

Some limitations were not avoidable in this study. First, this observational study collects patients from the single American Medical Center. The selection bias is not avoidable and the finding should be further confirmed by future studies in other medical centers from other countries. Second, some confounding factors influencing the correlation between thrombocytopenia and mortality may not be included such as platelet transfusion, coagulants transfusion. Third, the low number of baseline thrombocytopenia group would influence the reliability of our findings, which should be verified by future studies with larger sample sizes. Fourth, only the occurrence and timepoints of thrombocytopenia were analyzed but not the severity of thrombocytopenia. The severity of low platelet level may also influence the correlation between thrombocytopenia and mortality of ICH. Future studies should be designed to further analyze the comprehensive effect of severity and timepoint of thrombocytopenia on the prognoses of ICH patients. Fifth, prognostic effect of thrombocytopenia on outcome was analyzed while the effect of correcting thrombocytopenia by platelet transfusion was not analyzed due to the inherent limitation of observational study. Randomized controlled studies are worthy to confirm therapeutic effect of platelet transfusion at different timepoints of hospitalizations on platelet level and prognosis. Finally, other outcomes such as functional status and rebleeding events of ICH patients were not analyzed due to the incomplete record of the database. Future studies could be designed to verify relationships between these outcomes and thrombocytopenia. Although these limitations, the strength of our study is exploring the diverse prognostic effect of the thrombocytopenia at different timepoints among ICH patients after admission.

## 5 Conclusion

Initial thrombocytopenia at admission is not associated with the mortality of ICH patients. ICH patients experiencing both initial thrombocytopenia and acquired thrombocytopenia have significantly higher mortality risk. The platelet level of ICH patients should be monitored constantly during hospitalizations to detect the thrombocytopenia and identify high mortality risk.

## Data Availability

Publicly available datasets were analyzed in this study. This data can be found here: mimic.physionet.org.
